# Comparing PrEP initiation rates by service delivery models among high risk adolescent boys and young men in KwaZulu-Natal, South Africa: findings from a population-based prospective study

**DOI:** 10.1186/s12889-024-18660-1

**Published:** 2024-04-24

**Authors:** Mbuzeleni Hlongwa, Wisdom Basera, Edward Nicol

**Affiliations:** 1 Public Health, Societies and Belonging, Human Sciences Research Council, Pretoria, South Africa; 2 School of Nursing and Public Health, University of KwaZulu-Natal, Durban, South Africa; 3 Burden of Disease Research Unit, South African Medical Research Council, Cape Town, South Africa; 4 School of Public Health and Family Medicine, University of Cape Town, Cape Town, South Africa; 5 Division of Health Systems and Public Health, Stellenbosch University, Cape Town, South Africa

**Keywords:** PrEP, PrEP initiation rates, Service delivery models, Men, KwaZulu-Natal, South Africa

## Abstract

**Introduction:**

Pre-exposure prophylaxis (PrEP) is an HIV prevention strategy that can reduce the risk of HIV acquisition by more than 90% if taken consistently. Although South Africa has been implementing PrEP since 2016, initially for selected population groups before expanding access to more people, there is a dearth of research focused on PrEP among adolescent boys and young men (ABYM), despite them experiencing high rates of HIV infection. To address this gap, we compared PrEP initiation rates by service delivery points (SDPs) among ABYM in KwaZulu-Natal, South Africa.

**Methods:**

We conducted a population-based prospective study in 22 SDPs from July 2021 to July 2022 in KwaZulu-Natal, South Africa. Sexually active ABYM aged 15–35 years who tested HIV negative were recruited at purposively selected PrEP SDPs (i.e., healthcare facilities, secondary schools and Technical Vocational Education and Training (TVET) colleges, and community-based youth zones). We collected baseline quantitative data from each participant using self-administered electronic questionnaires built into REDCap, including demographic information such as age, sex, employment status and level of education, as well as PrEP initiation outcomes. We extracted data from REDCap and exported it to Stata version 17.0 for analysis, and then eliminated discrepancies and removed duplicates. We described baseline characteristics using summary and descriptive statistics (median, interquartile range [IQR] and proportions) and reported PrEP initiation proportions overall and by SDPs.

**Results:**

The study included 1104 ABYM, with a median age of 24 years (interquartile range (IQR): 21–28)). Almost all participants were black African (*n* = 1090, 99%), with more than half aged 15–24 years (*n* = 603, 55%) and 45% (*n* = 501) aged 25–35 years. The majority (*n* = 963; 87%) had attained a secondary level of education. Overall PREP initiation rate among adolescent boys and young men was low: among 1078 participants who were eligible for PrEP, 13% (*n* = 141) were started on PrEP. Among the participants who were initiated on PrEP, over three quarters (78%, *n* = 58) were initiated from high schools, compared with community-based youth zones (40%, *n* = 37), TVET colleges (26%, *n* = 16) and healthcare facilities (4%, *n* = 30).

**Conclusions:**

This study provided evidence suggesting that expanding PrEP services to non-traditional settings, such as high schools, TVET colleges, and community-based organizations, may have a potential to increase PrEP access among ABYM in South Africa.

## Introduction

Human immunodeficiency virus (HIV) continues to be a significant public health concern in many parts of the world, consisting of more than 37 million people diagnosed with HIV in year 2020 [1]. South Africa has a high HIV prevalence, with more than seven million people estimated to be living with HIV [2]. Adolescent boys and young men (ABYM) are a vulnerable group affected by HIV in South Africa, as they account for a substantial proportion of people newly infected with HIV in South Africa [3]. In South Africa, ABYM are increasingly getting recognised as a vulnerable population group due to the disproportionately high burden of HIV infection, underscoring the urgent need to address the risk factors, including high-risk sexual behaviours and prioritizing AYBM in HIV prevention strategies. Pre-exposure prophylaxis (PrEP) is an HIV prevention strategy that can reduce the risk of HIV acquisition by more than 90% if taken consistently [4, 5]. Although this is the case, ABYM continue to experience high rates of HIV infection in South Africa, despite many efforts designed to address their HIV prevention care and needs [6]. HIV prevalence is 3.1 among males aged 15–19, 4.0 in 20–24 years, 6.3 in 25–29 years, 10.8 in 30–34 years and 16.9 in 35–39 years [7, 8]). Data from the HIV incidence study conducted in uMgungundlovu, and published in 2019 showed that among men aged 15–19, 20–24, 25–29 and 30–35 the HIV incidence rates were 0.24, 1.18, 2.84 and 1.45 per 100 person-years, respectively [7]. In South Africa, the rollout of oral PrEP started in 2016 with 11 sites in five provinces. An additional site was added in November, to create a total of 12 implementing sites in 2016 [9, 10]. Initially, PrEP was implemented among selected key population groups (for example, sex workers) as well as to men who have sex with men; and adolescent girls and young women at primary healthcare facilities, before expanding access to more people, including ABYM. However, there is a dearth of research focused on PrEP among ABYM, despite them experiencing high rates of HIV infection.

In order to improve the rates of PrEP initiation among ABYM, it is imperative that we understand which service delivery models may be effective to ensure that men have appropriate access to PrEP services. It is also important that we understand which service delivery models may contribute to low PrEP initiation rates. One potential factor for PrEP initiation is the service delivery model used to provide PrEP services. For example, traditional facility-based healthcare models may not effectively reach ABYM, due to several barriers men face to accessing healthcare services, including stigma, lack of privacy, long waiting queues, unfriendly healthcare environments and concerns about confidentiality [11, 12]. These challenges facing public healthcare facilities have led to the growing appetite for alternative models, including decentralizing models, that are aimed at improving the rates of PrEP initiation, such as community-based service delivery models. Community-based service delivery models may be more acceptable and accessible to AYBM compared to the traditional public healthcare facility-based models [13]. Research demonstrates that community-based ART service delivery and initiation are successful approaches for increasing HIV treatment access in SSA because they address a number of distinctive barriers related to receiving HIV treatment from clinic settings, including stigma and discrimination, long waiting times, confidentiality concerns, and transport costs [11, 14–16].

This study aimed to compare PrEP initiation rates by service delivery models among ABYM in KwaZulu-Natal, South Africa. Our study targeted adolescent boys and young men, a population that is at a greater risk for new HIV infections [6]. Specifically, this study assessed PrEP initiation rates among ABYM who accessed PrEP through high schools, technical and vocational education and training (TVET) colleges, community-based youth zones (i.e., spaces within communities dedicated to young people providing services focused on sexual and reproductive health and HIV-related services), and public healthcare facilities.

## Methods

### Study setting

**Fig. 1:**
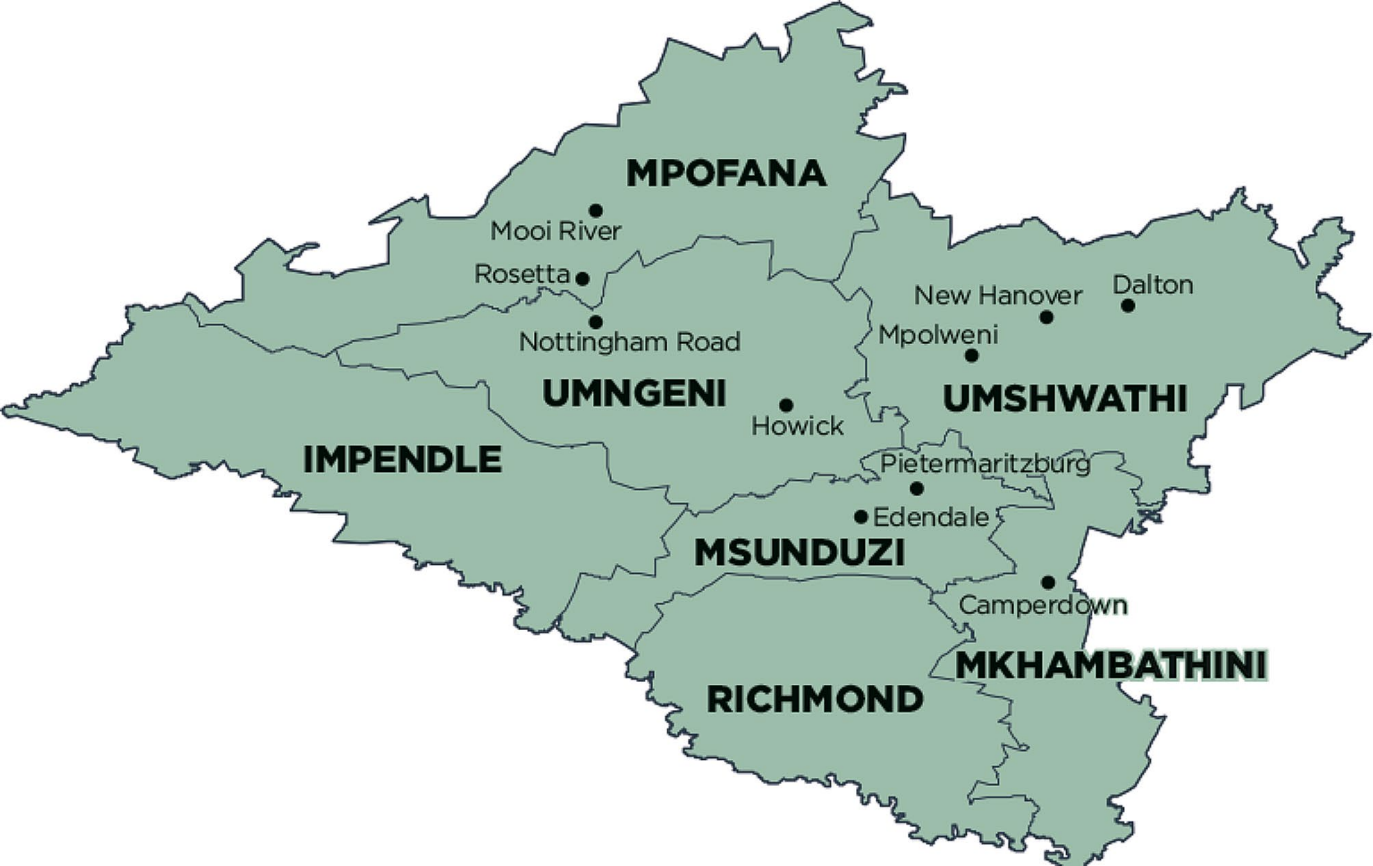
Sub-districts of uMgungundlovu District, KwaZulu-Natal, South Africa

This study was conducted in uMgungundlovu district in KwaZulu-Natal province, South Africa (Fig. 1). We selected participating facilities in consultation with provincial and district Department of Health. Overall HIV prevalence is high in uMgungundlovu district, accounting for 31% overall among 15–59 years, and 23% among men 15–49 years [17]. The population of uMgungundlovu is predominantly poor and rural, with the majority using public health services [18].

### Study design

We conducted a population-based prospective study in 22 SDPs over 13 months (July 2021 to July 2022). Adolescent boys and young men were purposively selected (those who visited the SDP while study staff were around) from 22 service delivery points (SDPs) (i.e., healthcare facilities (*n* = 11), High schools (*n* = 2) and Technical Vocational Education and Training (TVET) colleges (*n* = 4), and community-based youth zones(*n* = 5). We defined PrEP initiation according to national guidelines [10]. We administered questionnaires and accessed routine healthcare service records at enrolment to obtain and verify HIV test results and PrEP initiation outcomes among participants. The protocol for this study has been previously published [19].

### Population and recruitment

The target population for this study comprised sexually active ABYM aged 15–35 years, who test HIV negative during the routine healthcare facility-based, school-based, and community-based HIV testing services at 22 selected SDPs, in each of the seven sub-districts (strata) (namely, UMshwathi, Umgeni, Mpofana, Impendle, Msunduzi, Mkhambathini and Richmond) in the uMgungundlovu district. Participants were recruited and tracked through surveys using questionnaires and routine pharmaceutical records of PrEP pill collection, and SDP records. The inclusion criteria were: (a) having accepted an HIV test in one of the participating SDPs during the data collection, (b) having access to a cell phone and willingness to provide contact details, (c) be aged 15–35 years, (d) be sexually active and at high risk for HIV (identified by a set of risk assessment questions), (e) be seronegative based on HIV rapid test results on the day of recruitment, and (f) be able and willing to provide informed consent. Potential participants who were diagnosed with TB were excluded in the study.

### Data collection and management

Male clients that presented at the 22 SDPs for routine HIV test were approached before the test and recruited into the study. We collected baseline quantitative data from each participant using self-administered electronic questionnaires built into REDCap, including demographic information such as age, sex, employment status and level of education, as well as PrEP initiation outcomes, in addition to a HIV risk assessment test. Participants who had a negative HIV test and were substantially at risk were offered PrEP by staff at the SDPs. The risk assessment tool with the higher HIV risk stated in the parenthesis included a past six [6] months recall on (a) the number of people one had vaginal or anal sex with (2+), (b) frequency of condom use (no & don’t know), (c) reward based sexual experiences (yes), (d) having a sexually transmitted infection (yes & don’t know), (e) sharing of needles during intravenous drug use (yes) and (f) having a partner who is HIV infected (yes & don’t know). Any higher HIV risk response to one of the questions denoted PrEP eligibility. We also used secondary data sources, including the district health information system to extract information on PrEP initiation in the district and facilities where this project was implemented. Trained and competent facility-based data champions, working together with the project coordinator conducted daily quality checks on completeness of REDCap records on the tablets, before uploading data over 3G or Wi-Fi to the REDCap folder stored securely on the SAMRC’s server. Any data inconsistencies and/or errors were flagged, discussed and rectified at regular quality control meetings.

### Data analysis

We extracted data from REDCap and exported it to Stata version 17.0 for analysis, and then eliminated discrepancies and removed duplicates. We dropped 23 participants from our analysis because they did not have PrEP initiation outcomes.

We described baseline characteristics using summary and descriptive statistics (median, interquartile range [IQR] and proportions) and reported PrEP initiation proportions overall and by SDPs.

### Ethical considerations

The SAMRC Research Ethics Committee gave its clearance for this project (Ref: EC051-11/2020). Gatekeeper approvals were also obtained from the districts, facilities, and collaborating Provincial Departments of Health (Ref: KZ_202010_033). Prior to their involvement, we sought both verbal and written informed consent from all eligible study participants. Participants were informed throughout recruitment that participation was completely voluntary and that they were free to withdraw their participation at any time without facing any repercussions from the research team or the facilities they use.

## Results

**Fig. 2:**
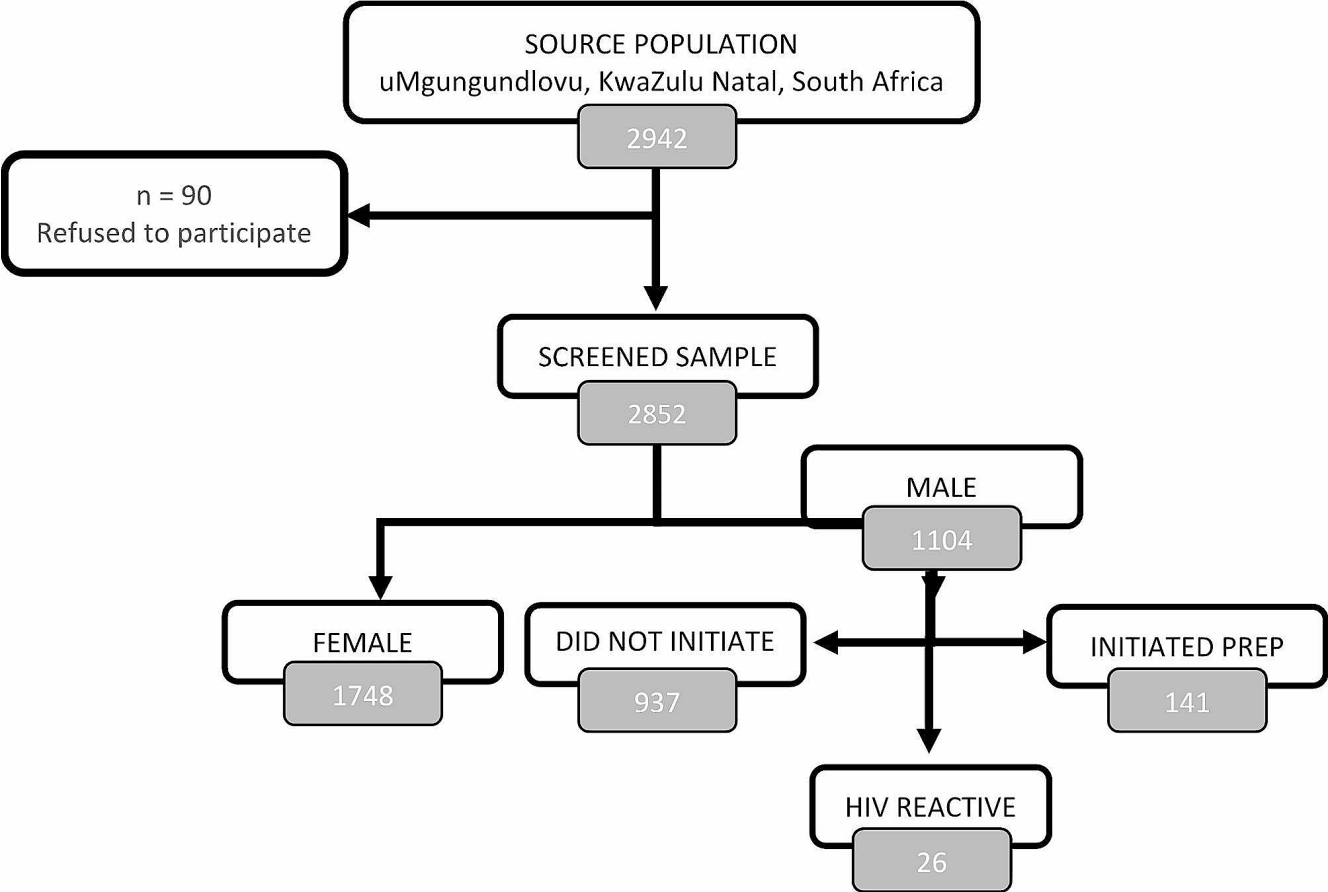
Consort diagram detailing the recruitment of study participants into the PrEP initiation in uMgungundlovu district

The study included 1104 ABYM recruited from 22 SDPs (Fig. 2), with a median age of 24 years (interquartile range (IQR): 21–28)) (Table 1). Almost all participants were black African (*n* = 1090, 99%), with more than half aged 15–24 years (*n* = 603, 55%) and 45% (*n* = 501) aged 25–35 years. The majority (*n* = 963; 87%) had attained a secondary level of education.

**Table 1:** Characteristics of adolescent boys and young men, KwaZulu-Natal, South Africa, 2022

Characteristic	Participants *n* = 1104
Age (years)	
Median (IQR)	24 (21–28)
Age categories, n (%)	
15–24	603 (55)
25–35	501 (45)
Population group, n (%)	
Black (African)	1090 (99)
Coloured (Asian)	10 (1)
Indian	1 (0)
Other (not specified)	3 (0)
Level of education	
No education	1 (0)
Primary	16 (1)
Secondary	963 (87)
Tertiary	118 (11)
No response	6 (1)
Employment status	
Unemployed	133 (12)
Employed	417 (38)
Studying	93 (8)
Other (not specified)	461 (42)

### PrEP initiation rates among adolescent boys and young men

Overall, PrEP initiation rate among adolescent boys and young men was low: among 1078 participants who were eligible for PrEP, 13% (*n* = 141) were started on PrEP (Table 2). Twenty-six (2%) participants were diagnosed with HIV, with 89% (*n* = 23) reactive participants coming from clinics and 11% (*n* = 3) from TVET colleges.

**Table 2:** PrEP initiation rates (*n* = 1078) by service delivery models among adolescent boys and young men, KwaZulu-Natal, South Africa, 2022

Service delivery model	Initiated on PrEP, n (%)	Not initiated on PrEP, n (%)	Total, n (%)
Clinics	30 (4)	820 (96)	850 (100)
Youth zones	37 (40)	55 (60)	92 (100)
TVET colleges	16 (26)	46 (74)	62 (100)
High schools	58 (78)	16 (22)	74 (100)
Total	141 (13)	937 (87)	1078 (100)

Another notable finding in our study is the disparities in PrEP initiation rates among different types of service delivery points. For example, among the participants who were initiated on PrEP, over three quarters (78%, *n* = 58) were initiated from high schools, compared with community-based youth zones (40%, *n* = 37), TVET colleges (26%, *n* = 16) and healthcare facilities/clinics (4%, *n* = 30).

Over three quarters (79%, *n* = 849) of the participants had a high risk profile of HIV infection based on the risk screening tool and were eligible for PrEP. A high proportion (61%, *n* = 661) of participants self-reported as willing to consider taking PrEP, with 16% (*n* = 107) of those who went on to initiate it. Amongst the reasons cited by those at a higher HIV risk profile (*n* = 849) for non willingness to consider PrEP, the top three were – I don’t want to be taking drugs for a long time (13%, *n* = 112), I fear side effects (11%, *n* = 91) and I do not think I am at risk of acquiring HIV (10%, *n* = 84).

## Discussion

This study aimed to compare PrEP initiation rates by service delivery models among ABYM in KwaZulu-Natal, South Africa. Similar to our study, PrEP initiation rates among men were reported to be low in Eswatini [20]. This supports the notion that PrEP access and uptake is a widespread challenge in the region, given the several factors and barriers identified to be affecting men’s access and initiation to PrEP in SSA, including stigma, discrimination, lack of knowledge and awareness of PrEP, inaccessibility of PrEP services, misinformation, fear of side effects and PrEP pill burden [21–23]. These findings suggest that there is a need for targeted educational interventions aimed to promote PrEP awareness and improve PrEP initiation rates among ABYM in KwaZulu-Natal, and in similar settings elsewhere.

Specifically, our study found that healthcare facilities had lower rates of PrEP initiation compared to high schools and TVET colleges, and community-based youth zones. This result suggests that the traditional facility-based may not be the most effective approach for reaching adolescent boys and young men with PrEP services. Instead, our study findings suggest that the community-based youth zones and high schools and TVET colleges may be promising additional models to traditional healthcare facilities for reaching men through distributing PrEP services.

Our finding reporting low rates of PrEP initiation from healthcare facilities, compared to community-based youth zones and high schools and TVET colleges was not surprising, given the well documented barriers deterring men from accessing services from the healthcare facilities in SSA [11]. In South Africa, for example, compared with women, men are less likely to access healthcare services, due to many factors including stigma, long waiting queues, masculinity and unfriendly healthcare environments [12]. However, our findings are consistent with global efforts to decentralize HIV prevention and treatment services to community-based settings in an effort to improve access to HIV treatment.

Evidence shows that community-based HIV services are an effective strategy for improving access to HIV treatment in SSA, as it addresses several distinctive barriers associated with accessing HIV treatment from clinic settings [14–16]. Therefore, in order to address the low rates of PrEP initiation among ABYM in KwaZulu-Natal, our findings suggest that there may be a need to adopt a multi-pronged approach that may include targeted outreach and education interventions, as well as decentralizing PrEP services to non-traditional healthcare settings. This may include strengthening and expanding partnering with high schools and TVET colleges, as well as community-based organisations to provide education and counselling, as well as improving PrEP access for men. This could involve developing and/or strengthening tailored educational materials, peer-led counselling sessions, and targeted outreach initiatives to increase knowledge of PrEP among males who might not be currently receiving these services.

A key limitation for this study relates to the fact that this paper does not address the factors influencing PrEP initiation rates among ABYM. Instead, these are currently being analyzed qualitatively, and will be discussed in a separate publication.

## Conclusions

In conclusion, the findings from this study have important indications for efforts to improve PrEP access among ABYM in KwaZulu-Natal, South Africa. By expanding PrEP services to non-traditional settings, such as high schools, TVET colleges, and community-based organizations, there is potential to increase PrEP access and reduce the burden of HIV among men. These efforts may be particularly important in the context of the ongoing HIV epidemic in South Africa.

## Data Availability

The datasets used and/or analysed during the current study available from the corresponding author on reasonable request.
